# Maltase protein of *Ogataea (Hansenula) polymorpha* is a counterpart to the resurrected ancestor protein ancMALS of yeast maltases and isomaltases

**DOI:** 10.1002/yea.3157

**Published:** 2016-04-21

**Authors:** Katrin Viigand, Triinu Visnapuu, Karin Mardo, Anneli Aasamets, Tiina Alamäe

**Affiliations:** ^1^ Department of GeneticsInstitute of Molecular and Cell Biology, University of TartuEstonia; ^2^ Present address: Department of Systems BiologyTechnical University of DenmarkElektrovej, Building 3752800Kgs. LyngbyDenmark

**Keywords:** *α*‐glucosidase, maltase‐isomaltase, *Hp*MAL1, protein evolution, methylotrophic yeast, differential scanning fluorimetry

## Abstract

*Saccharomyces cerevisiae* maltases use maltose, maltulose, turanose and maltotriose as substrates, isomaltases use isomaltose, *α*‐methylglucoside and palatinose and both use sucrose. These enzymes are hypothesized to have evolved from a promiscuous *α*‐glucosidase ancMALS through duplication and mutation of the genes. We studied substrate specificity of the maltase protein MAL1 from an earlier diverged yeast, *Ogataea polymorpha* (*Op*), in the light of this hypothesis. MAL1 has extended substrate specificity and its properties are strikingly similar to those of resurrected ancMALS. Moreover, amino acids considered to determine selective substrate binding are highly conserved between *Op* MAL1 and ancMALS. *Op* MAL1 represents an *α*‐glucosidase in which both maltase and isomaltase activities are well optimized in a single enzyme. Substitution of Thr200 (corresponds to Val216 in *S. cerevisiae* isomaltase IMA1) with Val in MAL1 drastically reduced the hydrolysis of maltose‐like substrates (*α‐*1,4‐glucosides), confirming the requirement of Thr at the respective position for this function. Differential scanning fluorimetry (DSF) of the catalytically inactive mutant Asp199Ala of MAL1 in the presence of its substrates and selected monosaccharides suggested that the substrate‐binding pocket of MAL1 has three subsites (–1, +1 and +2) and that binding is strongest at the –1 subsite. The DSF assay results were in good accordance with affinity (*K*_m_) and inhibition (*K*_i_) data of the enzyme for tested substrates, indicating the power of the method to predict substrate binding. Deletion of either the maltase (*MAL1*) or *α*‐glucoside permease (*MAL2*) gene in *Op* abolished the growth of yeast on MAL1 substrates, confirming the requirement of both proteins for usage of these sugars. © 2016 The Authors. *Yeast* published by John Wiley & Sons, Ltd.

**Abbreviations**
*α*‐MG, *α*‐methylglucoside/methyl‐*α*‐d‐glucopyranoside
*α‐p*NPG, *p*‐nitrophenyl‐*α*‐d‐glucopyranoside
2‐DG, 2‐deoxy‐d‐glucose
DSF, differential scanning fluorimetry
*Hp*, *Hansenula polymorpha*
HPLC, high‐performance liquid chromatography
IMOs, isomalto‐oligosaccharides
*k*_cat_, catalytic constant
*K*_i_, inhibition constant
*K*_m_, the Michaelis constant
MAL1, maltase protein
*Op*, *Ogataea polymorpha*
TLC, thin‐layer chromatography
*T*_m_, melting temperature
WT, wild‐type

For abbreviations of names of yeast species, see supporting information, Table S2.

## Introduction

Redundant genes for the utilization of *α*‐glucosides reside in subtelomeric regions of *Saccharomyces cerevisiae* (*Sc*) chromosomes (Needleman, 1991; Naumov *et al.*, 1994). These genes may encode proteins, e.g. *α*‐glucosidases, with varied sequence and function (Brown *et al.*, 2010; Naumov and Naumoff, 2012; Voordeckers *et al*., 2012). *Sc* has two types of *α*‐glucosidase: *α‐*1,4‐glucosidase (EC 3.2.1.20, maltase) and oligo‐*α‐*1,6‐glucosidase (EC 3.2.1.10, isomaltase) (Yamamoto *et al.*, 2004), both belong to family 13 of the glycoside hydrolases (GH13), according to the CAZy classification (Lombard *et al*., 2014). Maltases use maltose, maltulose, turanose and maltotriose as substrates; isomaltases use isomaltose, *α*‐methylglucoside (*α*‐MG) and palatinose; whereas both use sucrose and *p*‐nitrophenyl‐*α*‐d‐glucopyranoside (*α*‐*p*NPG) (Yamamoto *et al*., 2004; Teste *et al.*, 2010; Voordeckers *et al*., 2012; Deng *et al*., 2014). Maltase genes and the respective proteins (Krakenaĭte and Glemzha, 1983; review by Needleman, 1991) were described earlier than those of isomaltase. Yamamoto *et al*. (2004) cloned the first isomaltase gene of *Sc* and studied the substrate specificity of the protein. Later, this gene was designated *IMA1* (Naumov and Naumoff, 2010; Teste *et al*., 2010). *IMA1* is a member of a novel family of *IMA* genes (*IMA1–IMA5*) discovered in the genome of *S. cerevisiae* S288C by phylogenetic analysis (Naumov and Naumoff, 2010), their identification was confirmed using functional genomic analysis by Teste *et al*. (2010).

Recently, maltases and isomaltases and their transcriptional activators were addressed from the aspect of protein evolution (Brown *et al.*, 2010; Voordeckers *et al*., 2012; Pougach *et al*., 2014). Voordeckers *et al.* (2012) hypothesized that *Sc* maltases and isomaltases have evolved from an ancestral promiscuous protein, ancMALS, through gene duplications and following evolution giving rise to specialized proteins, maltases and isomaltases. The whole‐genome duplication of ancestral *Saccharomyces* (Wolfe and Shields, 1997) agrees nicely with this hypothesis. A considerable sequence identity in conserved active‐site regions of isomaltases and maltases (Voordeckers *et al.*, 2012) also suggests their shared ancestry.

**Figure 1:**
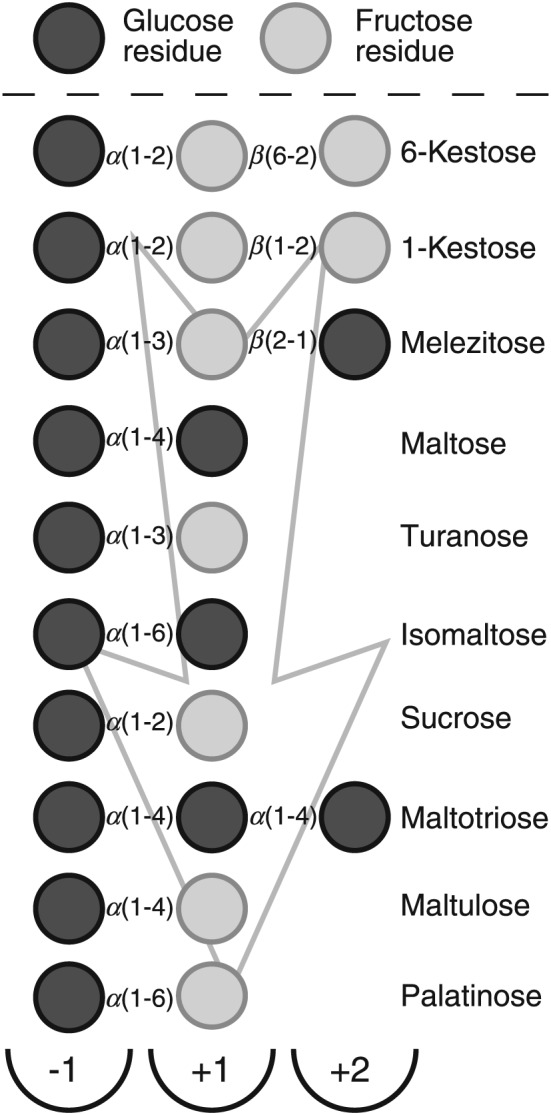
A scheme of composition of *Op* maltase substrates, with linkages between the sugar residues indicated, as also are subsites –1, +1 and +2 of the enzyme's substrate‐binding pocket that are expected to bind respective sugar residues; arrow, increase of the affinity of the enzyme towards the depicted substrate (data presented in Tables 1 and 2). Cleavage of the glycosidic linkage is executed between the residues bound at the –1 and +1 subsites

To test the hypothesis, the authors synthesized *in vitro* several *in silico*‐predicted ancient variants of *α*‐glucosidases and studied their substrate selection. Among the substrates tested on resurrected enzymes were maltose, sucrose, turanose, maltotriose and maltulose (maltose‐like sugars) and isomaltose, palatinose, *α*‐methylglucoside (methyl‐*α*‐d‐glucopyranoside; *α*‐MG) that are isomaltose‐like sugars. Details of the monomeric composition and linkage types of these sugars are given in Figure 1. Even though ancestral ancMALS could use both types of substrates, the authors considered that these two activities could not be fully optimized in a single (resurrected) enzyme (Voordeckers *et al.*, 2012).

**Table 1:** Preliminary evaluation of substrate specificity of *Op* maltase

Substrate	Specific activity (U/mg) at two substrate concentrations	
	50 mm	100 mm
*Maltose‐like*		
*α‐p*NPG^a^	78.4 ± 6.2 (1 mm)	97.4 ± 6.9 (2 mm)
Maltose^b^	77.8 ± 0.8	107.4 ± 3.5
Sucrose	115.9 ± 6.3	131.3 ± 2.3
Turanose	107.2 ± 4.5	133.4 ± 2.7
Maltotriose	145.5 ± 7.0	171.3 ± 9.5
Maltulose	23.3 ± 0.1	26.8 ± 1.3
1‐Kestose	17.1 ± 1.8	35.4 ± 5.5
Melezitose	17.7 ± 1.1	27.3 ± 0.4
*Isomaltose‐like*		
*α*‐MG	16.0 ± 0.9	19.3 ± 0.6
Isomaltose^b^	12.5 ± 0.4	13.6 ± 1.5
Palatinose (isomaltulose)	20.1 ± 1.0	21.1 ± 1.0

Specific activities (U/mg; μm glucose liberated/min × mg protein) were calculated from initial reaction velocities on 100 mm and 50 mm substrate in maltase buffer at 37°C. Protein content in reaction mixtures was 3.6–7.2 µg/ml; in the case of *α‐p*NPG as a substrate it was 0.14 µg/ml. SD values of two to four parallel measurements are shown.

a Substrate was used at 1 and 2 mm concentrations and specific activity was quantified according to release of *p*NP.

b The values of initial velocities were divided by two, due to the release of two glucose molecules/one substrate‐splitting reaction.

**Table 2:** Kinetic parameters of *Op* MAL1 and MAL1 mutant Thr200Val

Substrate	MAL1			MAL1 Thr200Val		
	*K*_m_ (mm) ± SD	*k*_cat_ (1/min) ± SD	*k*_cat_/K_m_ (1/mm/min) ± SD	*K*_m_ (mm) ± SD	*k*_cat_ (1/min) ± SD	*k*_cat_/K_m_ (1/mm/min) ± SD
Maltose‐like substrates						
*α‐p*NPG^b^	0.52 ± 0.05	8476.4 ± 297.8	16300.7	0.37 ± 0.03	4870.0 ± 255.0	13262.4
Maltose	51.8 ± 3.7	10395.3 ± 297.8	200.7	375.9 ± 26.1	2104.1 ± 64.3	5.6
Sucrose	25.1 ± 2.4	10984.2 ± 416.9	437.6	104.8 ± 10.1	5121.4 ± 709.1	48.9
Turanose	34.0 ± 2.6	11864.3 ± 370.5	348.9	157.3 ± 14.6	5713.6 ± 281.7	36.3
Maltotriose	20.2 ± 1.7	13597.9 ± 377.2	673.2	137.9 ± 8.8	4434.6 ± 150.1	32.2
Maltulose	15.8 ± 0.8	2038.0 ± 33.1	129.0	7.3 ± 0.7	596.1 ± 28.4	81.4
Melezitose	115.0 ± 8.2	3870.9 ± 145.6	33.7	10 000^a^	NA	NA
Isomaltose‐like substrates						
*α*‐MG	35.5 ± 3.3	1806.4 ± 66.2	50.9	49.3 ± 4.1	2342.4 ± 108.4	47.5
Isomaltose	27.3 ± 2.3	1191.1 ± 72.9	43.6	40.8 ± 4.0	2100.8 ± 103.9	51.5
Palatinose	6.8 ± 0.4	1495.4 ± 26.5	219.9	11.4 ± 0.47	2276.2 ± 29.1	200.0

a The value of *K*_m_ was too high to be measured accurately and was set to. 10 000.

b Specific activities were quantified according to release of *p*NP.

NA, not applicable; SD, standard deviation of the mean value.

Our data present more evidence to consider a protein similar to ancMALS to be a plausible ancestor of the isomaltases and maltases of modern‐day yeasts. We describe here the MAL1 protein of *Ogataea polymorpha* (*Op*), which is highly similar to resurrected ancMALS variants but which has not been characterized in detail before. *Op* belongs to the yeasts that have diverged from the main line of evolution earlier than *Sc* (Kurtzman *et al.*, 2015). Voordeckers *et al.* (2012) used the *Op* maltase protein sequence (GI: 7739797) in alignments and sequence analysis, but did not address the catalytic properties of the protein.

We cloned the maltase gene *MAL1* of *Op* about 15 years ago (Liiv *et al.*, 2001) and later studied its regulation (Alamäe *et al.*, 2003; Suppi *et al.*, 2013). A study of crude cell extracts of *MAL1*‐expressing *E. coli* (Liiv *et al.*, 2001) showed that the *Op* maltase hydrolysed not only maltose and sucrose but also *α*‐MG (for structure, see supporting information, Figure S1), which is a synthetic analogue of isomaltose and a substrate for isomaltases (Yamamoto *et al*., 2004; Teste *et al.*, 2010). The growth ability of *Op* wild‐type (WT) strain and a maltase deletant on different sugars indicated that *Op* maltase must also use maltotriose and turanose (Alamäe *et al.*, 2003; Viigand and Alamäe, 2007). These features hinted that MAL1 of *Op* may have wide substrate specificity, and that it could also use natural isomaltose‐type substrates.

Here we studied the substrate specificity profile of MAL1 in more detail, comparing our results with data available for resurrected proteins and existing *α*‐glucosidases of yeasts, and also paying attention to the phylogenesis of these enzymes and yeasts. We studied the activity of purified *Op* MAL1 on nine substrates used by Voordeckers *et al.* (2012), but also tested some additional oligosaccharides (kestoses and nystose) and oligosaccharide mixtures (malt extract and isomalto‐oligosaccharides) as potential substrates for the maltase. To assess binding of the substrates to the active site, we constructed catalytically inactive mutant Asp199Ala (D199A) of MAL1 and performed differential scanning fluorimetry (DSF) of the protein in the presence of *α*‐glucosides and some selected monosaccharides as ligands. We also studied the importance of Thr200 (corresponds to Val216 in isomaltase IMA1; Yamamoto *et al.*, 2004) in the substrate selection of MAL1. Finally, we assayed the effect of deletion of maltase and *α*‐glucoside permease genes on the ability of respective mutants to grow on sugars that serve as substrates for the MAL1 protein.

## Materials and methods

### Yeast and bacterial strains, substrates, media and cultivation

*Ogataea polymorpha* HP201 (*leu2‐2 ura3‐1 met4‐220*) used in this study was obtained from Dr K. Lahtchev (Sofia, Bulgaria) and is maintained in the CELMS collection at the Institute of Molecular and Cell Biology (IMCB), University of Tartu, Estonia. The strain is isogenic to CBS4732 (Lahtchev *et al.*, 2002), which was earlier allocated to the species *Hansenula polymorpha*. Several sibling species in the *H. polymorpha* complex were described earlier (Naumov *et al*., 1997) and currently (Suh and Zhou, 2010; Naumova *et al*., 2013) the CBS4732 strain belongs to *Ogataea polymorpha*. *Op* wild‐type strain and respective deletion mutants of maltase and *α*‐glucoside permease genes (Alamäe *et al.*, 2003; Viigand and Alamäe, 2007) were grown in 0.67% yeast nitrogen base (YNB) medium (Difco, USA) without amino acids, with required auxotrophic supplements added; 2% agar was used for solid media. Sugars were sterilized by filtration and used at the concentrations shown below. *Escherichia coli* BL2 (DE3) (Studier and Moffatt, 1986) transformants were grown in Luria–Bertani (LB) medium containing 0.15 mg/ml ampicillin. Liquid cultures were aerated on a shaker. The cultivation temperature of *Op* and *E. coli* was 37°C. *Schizosaccharomyces pombe* (*Sp*) 972 h^–^ (ATCC 24843), kindly donated by Dr Tiina Tamm (University of Tartu, Estonia), was grown at 30°C. Sugars used for substrate specificity screening of the maltase and as carbon sources for the yeasts were: d‐maltose, sucrose, *α*‐MG, isomaltose, maltulose, d‐turanose, 1‐kestose, nystose, raffinose, d‐melezitose, maltotriose, *p*‐nitrophenyl‐*α*‐d‐glucopyranoside (*α‐p*NPG) and malt extract (all purchased from Sigma‐Aldrich, Germany) the palatinose (isomaltulose) sample was received as a gift from Beneo (Belgium). Isomalto‐oligosaccharides (IMOs) were from Wako Chemicals GmbH (Japan). D‐mannose, d‐galactose and 2‐deoxy‐d‐glucose (2‐DG) used in the DSF assay (see below) were from Sigma‐Aldrich (Germany). Acarbose and d‐panose were from Sigma‐Aldrich (Germany) and d‐glucose from Carl Roth GmbH (Germany). *Op* was grown on 0.2% sugars and *Sp* on 2% sugars, except for IMOs, which were used at 0.2% for both yeasts. Growth was evaluated on day 5 in the case of *Op* and on day 11 in the case of *Sp*.

### Cloning, site‐directed mutagenesis, heterologous expression and purification of *Op* maltase

The primers MAL1_PURICterm_Fw and MAL1_PURICterm_Rev (see supporting information, Table S1) were designed, according to the *Op* maltase gene *MAL1* sequence (GenBank: AAF69018.1; GI: 7739797), to amplify a 1692 bp product from pHIPX8MAL1 (Visnapuu *et al.*, 2008) for ligation‐free PCR‐based cloning (Curiel *et al.*, 2011; Visnapuu *et al*., 2011). The resulting plasmid was designated pURI3–MAL1Cter. Recombinant *Pfu* polymerase (Thermo Scientific, USA) was used in cloning procedures and site‐directed mutagenesis. The restriction endonucleases *Dpn*I and *Not*I were purchased from Thermo Scientific (USA).

Mutations were introduced into the *MAL1* gene by PCR using mutagenic primers and subsequent extension of the sequence on pURI3–MAL1Cter, similarly as in Visnapuu *et al.* (2011). Information on primers and codon changes is presented in Table S1 (see supporting information). DNA Clean & Concentrator™‐5 kit (Zymo Research, USA) was used for purification and concentration of the PCR products and DNA fragments. Plasmid DNA was purified using a FavorPrep™ Plasmid Extraction Mini Kit (Favorgen Biotech Corp., Taiwan) and the mutations were verified by DNA sequencing. Plasmids containing either WT or mutated *MAL1* gene were electroporated into *E. coli* BL2 (DE3) for heterologous expression. The maltase variants expressed from pURI3–MAL1Cter contain a His_6_ tag at their C‐termini, enabling their purification by Ni^2+^‐affinity chromatography. The purification of maltases and evaluation of the purity of preparation were performed essentially as in Visnapuu *et al.* (2011). To prevent precipitation of the purified protein, 300 mm NaCl was added to the dialysis buffer (100 mm K‐phosphate buffer, pH 6.5, 0.02% Na‐azide). Protein was quantified by measuring the absorbance of the solution at 280 nm. The respective extinction coefficients were computed at the ExPASy Proteomics Server (http://expasy.org). The purified protein was stable in the buffer, maintaining its catalytic activity at 4°C for several months.

### Determination of substrate specificity and kinetic parameters of the *Op* maltase

The substrate specificity of MAL1 was determined by measuring the release of glucose from the substrates, as in the case of the levansucrase reaction (Visnapuu *et al.*, 2011). Hydrolysis of *α‐p*NPG was assayed as in Liiv *et al.* (2001), according to release of *p*‐nitrophenol. In all enzyme activity measurements, 100 mm K‐phosphate buffer, pH 6.5, containing 0.1 mm EDTA (maltase buffer) (Liiv *et al.*, 2001) was used and the reactions were conducted at 37°C.

First, the ability of MAL1 to hydrolyse the substrates was assayed at two substrate concentrations, 50 and 100 mm. Protein was added at 3.6–7.2 µg/ml in the case of sugars as substrates and at 0.14 µg/ml in the case of *α‐p*NPG. At certain time points, aliquots were withdrawn, combined with three volumes 200 mm Tris buffer, pH 8.3, and subsequently heated at 96°C for 5 min to stop the reaction. The content of released glucose was determined spectrophotometrically, using Glucose Liquicolor Reactive (Human GmbH, Germany), as in Visnapuu *et al.* (2011). Specific activities (μm substrate cleaved/min × mg protein; U/mg) were calculated from the initial velocities of the reaction. For kinetic analysis, the initial rates of glucose release from substrates were measured at several concentrations of the substrate, as described above, conducting at least two independent measurements for each substrate and concentration. Sugars were used at four to six concentrations in the range 2.5–200 mm. Protein was added at 3.6–7.2 µg/ml in the case of WT MAL1 and 5.5–11.0 µg/ml for the Thr200Val mutant. The Asp199Ala mutant was only tested on 100 mm sugar concentrations with 247 µg/ml protein. In the *α‐p*NPG assay, protein was used at 0.14 µg/ml in the case of WT MAL1, 0.11 µg/ml for Thr200Val and 247 µg/ml for Asp199Ala mutants. The initial velocity data analysis using the enzyme kinetics module of SigmaPlot (Systat, USA) yielded kinetic parameters (*K*_m_, *V*_max_) for the enzymes; *k*_cat_ and *k*_cat_/*K*_m_ were calculated from these data. Theoretical *M*_w_ values of the proteins for *k*_cat_ calculation were computed in the ExPASy Proteomics Server (http://expasy.org). Maltase inhibition by monosaccharides and acarbose was studied by incubating the enzyme with 0.1–3.0 mm *α‐p*NPG in the presence of the following substrates: 0.05 mm acarbose, 20 mm d‐glucose, 400 mm d‐mannose, 400 mm d‐fructose, 400 mm 2‐DG or 400 mm d‐galactose. Before initiation of the reaction with *α‐p*NPG, the protein was pre‐incubated with the inhibitor for 5 min at 37°C in the case of acarbose, d‐glucose and d‐fructose and for 20 min in case of d‐mannose, 2‐DG and d‐galactose. The *K*_i_ values were calculated using the enzyme kinetics module of SigmaPlot (Systat, USA).

### Thin‐layer chromatography (TLC) of the reaction products

To verify substrate hydrolysis and detect the products formed, MAL1 WT and mutant Asp199Ala reaction mixtures were separated on TLC plates (Silica Gel 60 F_254_) with concentrating zones (Merck, Germany). The reactions were conducted in maltase buffer at 37°C, and at certain time points aliquots were withdrawn and heated for 5 min at 96°C to inactivate the enzyme. Concentrations of 1‐kestose, nystose, melezitose and maltotriose in the reaction mixture were 50 mm, 6‐kestose was used at 10 mm, and malt extract and IMOs were used at 2% w/v. Palatinose and maltose were used at 100 mm in the assay of catalytic ability of the Asp199Ala mutant. Maltase was added at 1 U/ml reaction mixture, or at 247 µg/ml in the case of the Asp199Ala mutant. The amount of protein equivalent to 1 U was determined through activity assays on 1 mm *α‐p*NPG; 0.5 µl of each of the stopped reaction mixtures were spotted onto TLC plates and sugars were separated with two runs in chloroform:acetic acid:water (6:7:1 v/v/v) (Stingele *et al.*, 1999). Sugars were visualized by immersion of the plates in aniline–diphenylamine reagent and subsequent heating of the dried plates at 120°C (Jork *et al.*, 1990).

### Thermostability assay of *Op* maltase in the presence and absence of sugars

Thermostability of MAL1 was determined using two different methods. For the activity‐based method, 5 µm (3.6 µg/ml) or 25 µm (18 µg/ml in case of incubation temperatures of 45–60°C) of the *Op* WT maltase protein were incubated in maltase buffer for 30 min at temperatures 20°C, 30°C, 40°C, 45°C, 50°C and 60°C. After cooling of the samples on ice, residual activity of the enzyme was determined according to the hydrolysis of 50 mm sucrose at 37°C. Every temperature point was assayed in quadruplicate. The activity measured after incubation at 20°C was taken as 100%.

Differential scanning fluorimetry (DSF; also Thermofluor^®^) assay was performed essentially as in Mardo *et al.* (2014), using a LightCycler480 System (Roche, Switzerland) and SYPRO^®^ Orange (Sigma‐Aldrich, Germany) as a fluorescent dye. The samples contained 2 µm protein (WT or catalytically inactive mutant Asp199Ala) and 5× SYPRO Orange in 100 mm HEPES buffer, pH 7.0, with 150 mm NaCl. The melting point (*T*_m_) was defined as the temperature at which half the protein in the sample became unfolded. The Asp199Ala mutant was assayed by DSF in the presence of nine substrates of the enzyme, as well as some monosaccharides such as d‐galactose, d‐mannose, 2‐DG, d‐glucose and d‐fructose (see Figure 5).

### *In silico* methods

Gene and protein sequences were withdrawn from web‐based databases (GenBank, UniProtKB/Swiss‐Prot) and the genome sequences were reached through Mycocosm (Grigoriev *et al.*, 2014). Details of protein sequences used in alignments are given in Table S2 (see supporting information). Phylogenetic trees of maltase proteins were built at http://phylogeny.lirmm.fr (Dereeper *et al.*, 2008), which uses the MUSCLE program (Edgar, 2004) for alignment of protein sequences and PhyML3.0 (Guindon *et al.*, 2010; Castresana, 2000) for phylogenetic analysis of the protein sequences. Advanced analysis mode was used and 14 conserved blocks from primary alignment were selected for further alignment to build a phylogenetic tree. The signature amino acid sequences were aligned using ClustalW (Larkin *et al.*, 2007) and the resulting phylogram was visualized using TreeDyn 198.3 (Chevenet *et al.*, 2006). Sequence identity values between the *α*‐glucosidases were determined using ClustalW alignment and are presented in Table S3 (see supporting information).

## Results and discussion

### Substrate specificity of *O. polymorpha* maltase

The *Op MAL1* gene was cloned and overexpressed in *E. coli* as described in Materials and methods. Although the original promoter of the *MAL1* gene is recognized and functional in *E. coli*, due to *σ*70‐like elements in it (Alamäe *et al.*, 2003; Visnapuu *et al.*, 2008), we here applied a versatile pURI plasmid‐based expression system (Curiel *et al.*, 2011) to achieve superior expression.

First, we compared specific activities of purified *Op* maltase using 50 and 100 mm concentrations of substrates; a chromogenic substrate, *α‐p*NPG, was used at lower concentrations, due to enhanced affinity of the enzyme towards it. The substrates presented in Table 1 are grouped as maltose‐like (mainly *α‐*1,4 linkages, but may also contain *α‐*1,2 and *α‐*1,3 linkages; see Figure 1) and isomaltose‐like (with *α‐*1,6 linkages), similarly as in Voordeckers *et al.* (2012). According to the activity on 50 mm maltose‐like sugars, the substrate preference of MAL1 decreased in the order: maltotriose>sucrose>turanose>maltose>maltulose>melezitose>1‐kestose. Among isomaltose‐like substrates, palatinose was the most suitable one, followed by *α*‐MG and isomaltose. At 100 mm substrate concentration, the activities on maltose, melezitose and 1‐kestose increased, suggesting lower affinity of *Op* maltase for these substrates. A synthetic chromogenic substrate, *α‐p*NPG (see supporting information, Figure S1), is also a highly suitable substrate for MAL1 and the affinity of *Op* MAL1 for *α‐p*NPG was earlier shown to be around 0.5 mm (Alamäe and Liiv, 1998; Liiv *et al.*, 2001), which coincides with the current results (see Table 2).

**Figure 2:**
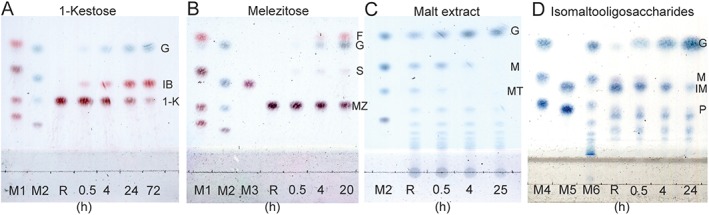
Time course of reaction of *Op* maltase with (A) 50 mm 1‐kestose, (B) 50 mm melezitose, (C) 2% malt extract and (D) 2% isomalto‐oligosaccharides, analysed by TLC. The reaction was conducted in maltase buffer, using 13 µg/ml maltase protein (1 U/ml): from top to bottom, M1, fructose, sucrose, 1‐kestose, nystose; M2, glucose, maltose, maltotriose, stachyose; M3, turanose; M4, glucose, maltose, maltotriose; M5, isomaltose, panose; M6, malt extract. References (R) designate (A) 50 mm 1‐kestose; (B) 50 mm melezitose; (C) 2% malt extract; and (D) 2% isomalto‐oligosaccharides (IMOs), respectively, without the enzyme added; G, glucose; IB, inulobiose; 1‐K, 1‐kestose; F, fructose; S, sucrose; MZ, melezitose; M, maltose; MT, maltotriose; IM, isomaltose; P, panose

Table 1 shows that 1‐kestose (Figure 1), which has not previously been assayed as a substrate for *α*‐glucosidases, is certainly cleaved by MAL1. TLC analysis (Figure 2A) revealed glucose and inulobiose [*β*‐d‐fructosyl‐(1 → 2)‐*β*‐d‐fructose] as reaction products, which was also confirmed by HPLC (data not shown). Additionally, MAL1 hydrolysed 6‐kestose (Figure 1) to glucose and levanbiose [*β*‐d‐fructosyl‐(2 → 6)‐*β*‐d‐fructose] (data not shown), indicating that the sucrose moiety is hydrolysed in both types of kestose. Yet a tetrasaccharide, nystose [*O‐α*‐d‐glucosyl‐(1 → 2)‐*β*‐d‐fructose‐(1 → 2)‐*β*‐d‐fructose‐(1 → 2)‐*β*‐d‐fructose] was not hydrolysed by MAL1 (see supporting information, Figure S2). To our knowledge, this is the first report showing that 1‐ and 6‐kestoses are substrates for a maltase. Therefore, at least the *Op* maltase can be used to modify fructo‐oligosaccharide mixtures containing kestoses (Visnapuu *et al.*, 2015). Maltase treatment should strip off glucose residues from 1‐ and 6‐kestoses, yielding inulo‐ and levanbiose, respectively. These short fructo‐oligosaccharides act as prebiotics – they are selectively metabolized by beneficial gut bacteria (Adamberg *et al.*, 2014; Visnapuu *et al.*, 2015).

Figure 2B shows that the primary products of melezitose (Figure 1) hydrolysis are sucrose and glucose, indicating enhanced affinity towards the turanose moiety of melezitose. At prolonged reaction times, released sucrose is further hydrolysed to glucose and fructose. As maltotriose and isomaltose were good substrates for the *Op* maltase protein (Tables 1 and 2), we analysed malt extract (which contains glucose, maltose, maltotriose and malto‐oligosaccharides) and commercial isomalto‐oligosaccharides (IMOs) as MAL1 substrates, using TLC (Figure 2C, D).

Figure 2C shows that maltose and maltotriose are both substrates for *Op* maltase. HPLC analysis showed that maltotetraose hydrolysis also occurred to a small extent, but longer oligomers (G_5_ and higher) were not hydrolysed (data not shown). From this aspect, *Op* maltase seems to differ from some other *α*‐glucosidases/maltases of yeast origin. For example, an extracellular *α*‐glucosidase of *Sp* uses a wide variety of malto‐oligosaccharides, including maltotetraose, ‐pentaose, ‐hexaose and ‐heptaose (Okuyama *et al.*, 2005). Notably, glucose released from maltose and maltotriose certainly inhibits the enzyme (Table 3). However, in a living yeast cell, glucose released from *α*‐glucosides is probably rapidly metabolized, decreasing the inhibitory effect on the maltase *in vivo*. According to our experiments, maltotriose is a better substrate for *Op* maltase than maltose (Tables 1 and 2) and, at least at the beginning of the reaction on maltotriose, glucose is stripped off and the resulting maltose is retained to be used later (data not shown). A similar situation was revealed in the case of malt extract hydrolysis: during the first 4 h of incubation, the amount of maltotriose decreased significantly, while maltose was still present (Figure 2C). IMOs also served as substrates for MAL1; release of glucose from these oligosaccharides was already evident at 30 min of reaction (Figure 2D). The latter figure also shows preferential hydrolysis of maltose over isomaltose. The hydrolysis of panose [*O‐α*‐d‐glucopyranosyl‐(1 → 6)‐*O‐α*‐d‐glucopyranosyl‐(1 → 4)‐d‐glucose] became evident after 4 h of reaction with *Op* MAL1.

**Table 3:** Inhibition of *α‐p*NPG hydrolysis by the *Op* maltase MAL1 in the presence of different substrates; respective literature data on *S. cerevisiae* maltase are shown for comparison

Inhibitor	*K*_i_ (mm) of *Op* maltase	*K*_i_ (mm) of *Sc* maltase	Reference for data on *Sc* maltase
Acarbose	0.0065 ± 0.0008	0.0778	Kim *et al.*, 1999
d‐Glucose	5.1 ± 0.7	2.5–3.7	Krakenaĭte and Glemzha, 1983; Yao *et al.*, 2003
d‐Mannose	114.7 ± 8.1	120	Yao *et al.*, 2003
2‐DG	168.7 ± 15.7	735	Yao *et al.*, 2003
d‐Fructose	213.1 ± 16.1	88	Krakenaĭte and Glemzha, 1983
d‐Galactose	250.7 ± 15.1	160	Yao *et al*., 2003

### The substrate specificity of *Op* maltase protein is strikingly similar to that of resurrected ancMALS

To assess substrate selection by *Op* maltase in more detail, we measured the enzyme's kinetics at varied concentrations of 10 substrates to calculate the *K*_m_, *V*_max_, *k*_cat_ and *k*_cat_/*K*_m_ values for every substrate and to compare the substrate utilization patterns between various natural and synthetic enzymes (Voordeckers *et al.*, 2012). Table 2 presents kinetic data for *Op* maltase and the mutant Thr200Val.

**Figure 3:**
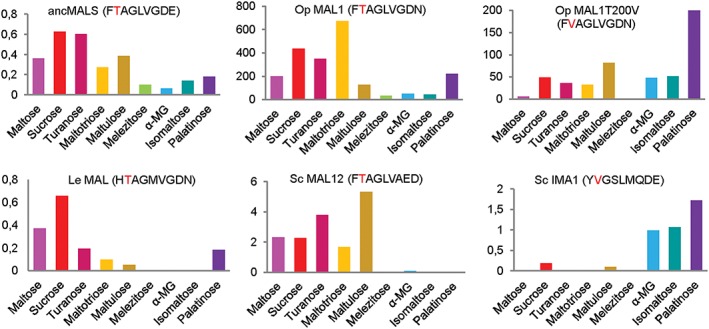
Catalytic efficiencies, *k*_cat_/*K*_m_ (mm/min) of *Op* maltase MAL1, ancient maltase ancMALS (G279), maltase of *Lodderomyces elongisporus (Le)*, maltase MAL12 of *S. cerevisiae* (*Sc*), isomaltase IMA1 of *Sc* and the T200V mutant of the *Op* MAL1. Data on other proteins, except for the *Op* MAL1 and its mutant, are taken from Voordeckers *et al.* (2012). Signiture amino acid sequence (see Figure 4) is presented with the residue corresponding to V216 (Val216) of *Sc* IMA1, shown in red. Mean values are presented; for standard deviation (SD) values, see Table 2 (this study) and Table S2 of Voordeckers *et al.* (2012)

Figure 3 compares the patterns of catalytic efficiency values of selected *α*‐glucosidases in the utilization of nine *α*‐glucosides. Some of these substrates were maltose‐like and some were isomaltose‐like (see Table 2). The resurrected ancMALS (G279) (Voordeckers *et al.*, 2012) was also included for comparison.

**Figure 4:**
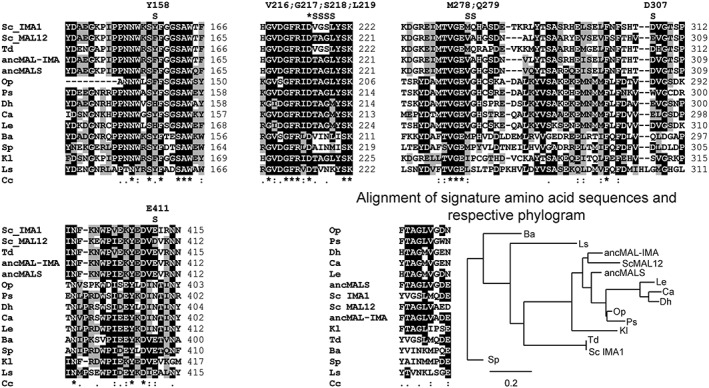
Sequence alignment blocks of 14 *α*‐glucosidases of various yeasts. Resurrected ancMALS (G279) and ancMAL‐IMA (A279) (Voordeckers *et al.*, 2012) are included in the alignment. Variable residues close to the active site (Yamamoto *et al.*, 2004, 2011; Voordeckers *et al.*, 2012) comprising the signature amino acid sequence are designated above the sequence (S); *position of the catalytic nucleophile (Asp215 in IMA1). A phylogram of enzymes resulting from MUSCLE alignment of signature amino acid sequences is also presented; scale below the phylogram indicates the number of substitutions/site. *Sc*, *S. cerevisiae* Sc288c; *Td*, *Torulapora delbrueckii*; *Op*, *Ogataea polymorpha*; *Ps*, *Pichia stipitis* PsAGL1; *Ca*, *Candida albicans*; *Dh*, *Debaryomyces hansenii*; *Le*, *Lodderomyces elongisporus*; *Ba*, *Blastobotrys* (*Arxula*) *adeninivorans*; *Sp*, *Schizosaccharomyces pombe*; *Kl*, *Klyuveromyces lactis*; *Ls*, *Lipomyces strarkeyi*; Accession Nos of sequences used and yeast strains are given in Table S2 (see supporting information)

The signature amino acid sequences presented in Figure 3 are compiled from the alignment of protein sequences (Figure 4) and include amino acid residues residing at positions that are predicted, or experimentally shown, to be crucial for specific binding of *α*‐glucosides to the enzyme (Yamamoto *et al.*, 2004, 2011; Voordeckers *et al.*, 2012; Deng *et al.*, 2014). Figure 4 shows protein sequence blocks extracted from a ClustalW alignment of 14 *α*‐glucosidases, including IMA1 of *Sc* and resurrected ancMALS (G279) and ancMAL‐IMA (A279) proteins. The positions comprising the signature amino acid sequence are designated above the alignments with ‘S’ and respective amino acid positions of *Sc* IMA1 are indicated.

Catalytic efficiencies of the maltases and isomaltases presented by Voordeckers *et al.* (2012) are much lower than of *Op* maltase (Figure 3). The respective values of *Sc* maltases and isomaltases reported by Krakenaĭte and Glemzha (1983), Yamamoto *et al.* (2011) and Deng *et al.* (2014) were also much higher compared to data by Voordeckers *et al.* (2012). However, as Voordeckers *et al.* (2012) produced and assayed an entire panel of proteins using the same experimental protocol, their study certainly enables comparison of the substrate utilization patterns between the enzymes. Importantly, the resurrected ancMAL‐IMA (A279) had higher catalytic efficiencies on all studied substrates when compared to ancMALS (G279) (Voordeckers *et al*., 2012), indicating that mixed substrate usage efficiency can be improved by *in vitro* protein evolution.

The catalytic efficiency patterns of *α*‐glucosidases (Figure 3) clearly exhibit IMA1 of *Sc* as a typical isomaltase and MAL12 as a typical maltase, the former preferring isomaltose‐like substrates and the latter maltose‐like ones. *Op* maltase uses both types of substrate, in this aspect being similar to ancMALS. Importantly, the signature amino acid sequences of *Op* maltase and ancMALS (G279) differ only in the last letter (Figures 3 and 4). The substrate specificities of resurrected ancMAL‐IMA (A279) and *Op* maltase are also quite similar, and their signature amino acid sequences differ by two letters (Figure 4). The respective signature sequences of ancMAL‐IMA (A279) (FTAGLVADE) and *Sc* MAL12 (FTAGLVAED) are also highly similar, but the substrate specificities of these enzymes largely differ: *Sc* MAL12 cannot hydrolyse isomaltose and palatinose (Voordeckers *et al.*, 2012). We consider that a residue in *α*‐glucosidases corresponding to E411 of IMA1 should be either N (asparagine) or E (glutamate) and not D (aspartate) to enable hydrolysis of isomaltose‐like sugars.

### Thr200Val substitution of *Op* MAL1 strongly decreases the cleavage of maltose‐like substrates by the enzyme, making it more similar to isomaltases

A Val residue (Val216 in the case of IMA1; Figure 4) in homology region II of *α*‐glucosidases was shown to be crucial in discriminating between the *α‐*1,4‐ and *α‐*1,6‐glycosidic linkages to be cleaved by the enzyme. A literature review by Yamamoto *et al.* (2004) cited that proteins having a Val at respective position hydrolysed either only the *α‐*1,6‐glucosidic linkage or both *α‐*1,4 and *α‐*1,6 linkages. At the same time, *α*‐glucosidases with a Thr at the respective position were specific for substrates containing *α‐*1,4 linkages. If Val216 in IMA1 was substituted with a Thr, the enzyme indeed gained the ability to hydrolyse maltose (Yamamoto *et al.*, 2004). We made a reverse mutation (from Thr to Val) of the respective position (Thr200) of *Op* MAL1. The Thr200Val mutant had largely decreased activity towards maltose, sucrose, turanose and maltotriose, and melezitose was no longer hydrolysed (Table 2). According to Table 2, the *K*_m_ values of the Thr200Val enzyme towards isomaltose‐like substrates were somewhat increased (affinity decreased), whereas the *k*_cat_ values were increased, resulting in practically unchanged values of catalytic efficiency. Data in Figure 3 and Table 2 show that, due to the Thr200Val mutation, the substrate utilization pattern of MAL1 became much more similar to that of isomaltases (e.g. *Sc* IMA1). Our data confirm that a Thr at the position equivalent to Val216 of IMA1 is required for efficient hydrolysis of maltose‐like substrates by *α*‐glucosidases. However, our results and data by Voordeckers *et al.* (2012) clearly show that *α*‐glucosidases that have a Thr at the position corresponding to Val216 of IMA1 can split not only *α‐*1,4 linkages in oligosaccharides but also many other types (*α‐*1,2, *α‐*1,3 and *α‐*1,6; see Figure 1). Comparison of protein sequences reveals a Thr at the respective position of typical maltases, such as *Sc* MAL32 and MAL12, as well as of promiscuous *α*‐glucosidases, such as ancMALS, and the respective enzymes of *Op*, *K. lactis* and *L. elongisporus* [see Figures 3 and 4 (this paper) and data in Voordeckers *et al.* (2012)]. Therefore, the substrate specificity of *α*‐glucosidases certainly has additional determinants. A mutational study of *Op* maltase may reveal interesting data on this issue.

### Differential scanning fluorimetry (DSF) of the catalytically inactive MAL1 protein as a tool to evaluate binding of the substrates to the enzyme

**Figure 5:**
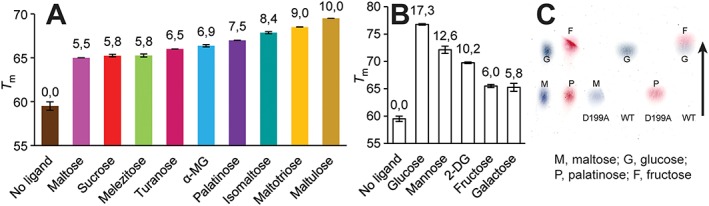
Thermostability of the catalytically inactive mutant D199A of *Op* MAL1 in the presence and absence of (A) the enzyme's substrates and (B) selected monosaccharides, both used at 300 mm concentration. *T*_m_ values for DSF assay of unliganded and liganded MAL1 are (A, B), with respective increases of *T*_m_ indicated above the bar. (C) TLC results of reactions of wild‐type MAL1 protein (WT; 1 U/ml added) or its catalytically inactive mutant D199A (added at 247 µg/ml) with maltose (a representative of maltose‐like substrates) and palatinose (a representative of isomaltose‐like substrates); arrow, direction of sugar separation on a TLC plate

There are no crystal structures available for maltases of fungal or yeast origin, and only one crystal structure is available for *Sc* isomaltase IMA1 (Yamamoto *et al.*, 2010, 2011). The catalytically active IMA1 was co‐crystallized with its competitive inhibitor maltose (Yamamoto *et al.*, 2010). For crystallization of IMA1 in complex with its natural substrate, isomaltose, a mutant Glu277Ala was constructed and used. In IMA1, Glu277 is a general acid–base catalyst and Asp215 is a catalytic nucleophile (Yamamoto *et al.*, 2011). Mutant Glu277Ala did not react with either maltose or isomaltose and its substrate‐bound structure indicated contacts between isomaltose and active site pocket amino acids (Yamamoto *et al.*, 2011). We constructed a catalytically inactive mutant of *Op* MAL1 by substituting the predicted catalytic nucleophile Asp199 with Ala. The Asp199Ala (D199A) mutant was catalytically inactive on all tested substrates used by the wild‐type enzyme (data not shown). Figure 5C shows hydrolysis of maltose and palatinose by the wild‐type maltase of *Op* and its absence in the case of the D199A mutant.

The D199A mutant can potentially be co‐crystallized with a variety of its native substrates. We conducted a DSF assay to evaluate the ability of potential ligands – enzyme substrates and potential inhibitors – to stabilize the protein in their presence. The *T*_m_ of the wild‐type maltase of *Op*, measured at pH 7.0 using DSF, was 51°C. This value was higher compared to respective data on *Sc* isomaltases in the range 36.3–46.6, pH 7.0, for wild‐type enzymes (Deng *et al.*, 2014). A classical thermostability assay indicated that catalytic activity of the *Op* maltase started to decrease after incubation at temperatures > 40°C. A 30 min incubation of MAL1 at 40°C reduced its catalytic activity to ~43% of the initial, and the same incubation time at 45°C to ~0.2% (data not shown). So, the *Op* maltase has intermediate thermostability, yet it is certainly more stable than *Sc* isomaltases. This can be explained by the thermophilic nature of *Op* (Kurylenko *et al.*, 2014). Quite surprisingly, the D199A variant of MAL1 had enhanced thermostability – its *T*_m_ in a DSF assay was close to 60°C. Importantly, enhanced stability of the D199A protein most probably increases its crystallization efficiency (Dupeux *et al.*, 2011). DSF assay of the D199A mutant in the presence of 300 mm substrates showed further thermostability increase being most prominent in the case of maltulose as a ligand, with the *T*_m_ value increased by 10°C (Figure 5A). In the presence of maltotriose, isomaltose and palatinose respective *T*_m_ values were increased by 9.0°C, 8.4°C and 7.5°C (Figure 5). Table 2 shows that the *K*_m_ values of WT maltase for maltotriose, maltulose and palatinose are quite low, which coincides with their good capacity to bind and stabilize the protein. Interestingly, the affinity for maltulose of the Thr200Val mutant was even higher (7.3 mm) than that of the wild‐type MAL1 (15.8 mm) (Table 2). The smallest stability increase (*T*_m_ elevated only by 5.5–5.8°C) was recorded for maltose, sucrose and melezitose as ligands (Figure 5). Regarding these substrates, affinity for maltose of MAL1 is rather low (Table 2). Low affinity for maltose has also been reported for *Sc* maltase (Krakenaĭte and Glemzha, 1983). As maltotriose is a perfect substrate for the enzyme (Table 2, Figures 1 and 3), we suggest that the substrate binding pocket of *Op* MAL1 has at least two + subsites, +1 and +2 (see Figure 1). Maltotriose also showed a perfect thermostabilizing ability of the maltase in a DSF assay. A trisaccharide, melezitose (Figure 1), also served as a substrate and its presence enhanced the thermostability of the D199A protein (Tables 1 and 2, Figure 5). According to the literature, multiple inhibition analysis also revealed at least three monosaccharide binding subsites for the *Sc* maltase protein (Yao *et al.*, 2003).

### DSF allows evaluation of binding of monosaccharide units to MAL1

To obtain further information on substrate binding, we assayed the thermal stability of MAL1 D199A in the presence of d‐glucose, d‐fructose, 2‐DG, d‐mannose and d‐galactose as ligands, all used at 300 mm concentration. The presence of glucose elevated the *T*_m_ value considerably, by 17.3°C, whereas the presence of 2‐DG by 10.2°C and fructose only by 6.0°C (Figure 5B). According to our interpretation, glucose enhances the stability of D199A most strongly, because it probably binds to all subsites of the enzyme, and binding at –1 is suggested to be the strongest, as it is a glucose‐specific subsite (Figure 1). Fructose most probably binds only at a +1 subsite and much less strongly (Figure 1). We assume that although 1‐kestose is a considerably good substrate for MAL1 (6‐kestose is a poor substrate), these kestoses should bind only via –1 and +1 subsites. Rather loose binding of a sugar residue at a +1 subsite was also shown for IMA1 protein, in which mostly hydrophobic contacts are involved (Yamamoto *et al.*, 2011).

2‐DG has no hydroxyl at C2. This particular hydroxyl is involved in hydrogen bonding of a non‐reducing glucose residue of isomaltose with Arg213 and Asp352 at the –1 subsite of IMA1 (Yamamoto *et al.*, 2011). We assume that the respective positions, Arg197 and Asp338 in *Op* maltase, are also important for the binding of glucose residues at the –1 subsite. We also tested d‐mannose (glucose epimer at C2) and d‐galactose (glucose epimer at C4) as MAL1 ligands in a DSF assay. D‐mannose stabilized MAL1 D199A well by elevating the *T*_m_ value by 12.6°C, suggesting that the active site of MAL1 can accommodate this sugar. The presence of d‐galactose increased the thermostability of MAL1 D199A much less (Figure 5B). Interestingly, Yao *et al.* (2003) showed, in an enzyme inhibition assay, that *Sc* maltase had a binding site for both of these sugars, yet they could not bind in a productive manner. We then investigated whether and how strongly d‐glucose, 2‐DG, d‐fructose, d‐mannose and d‐galactose inhibit the hydrolysis of *α‐p*NPG. Alongside with monosaccharides, inhibition by acarbose (a proven inhibitor of *α*‐glucosidases and an anti‐diabetic drug) was assayed. Table 3 shows that acarbose is a very strong competitive inhibitor of MAL1, with a very low *K*_i_ (6.5 µm) value. The respective value of *Sc* maltase was slightly higher (77.8 µm) and inhibition was also competitive (Kim *et al.*, 1999). Acarbose has been shown to inhibit *Sc* IMA1 somewhat less than *Sc* maltase (Deng *et al.*, 2014). Hydrolysis of *α‐p*NPG by MAL1 was competitively inhibited also by d‐glucose, 2‐DG, d‐fructose, d‐mannose and d‐galactose, with the strongest inhibition exerted by glucose (Table 3).

### *Op* maltase among yeast maltases and isomaltases: phylogenetic position and substrate specificity

**Figure 6:**
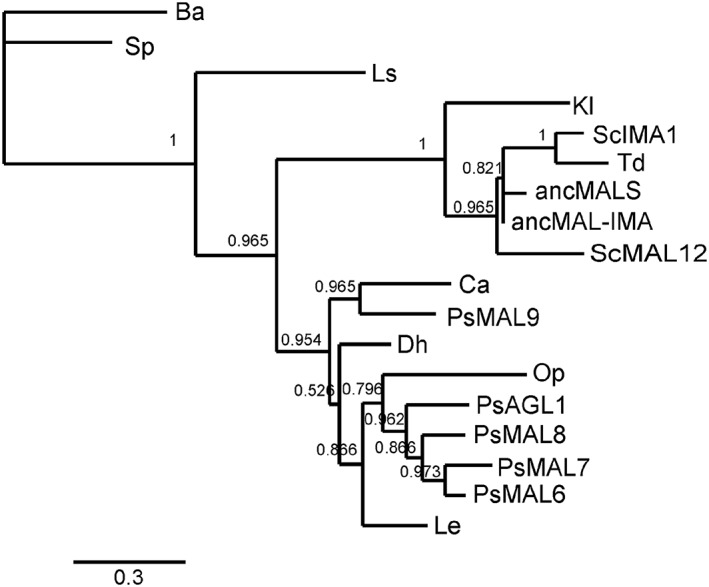
Phylogram of 18 *α*‐glucosidases of yeasts, constructed using the interface http://phylogeny.lirmm.fr. Bootstrap values are shown at the nodes; scale shows number of substitutions/site. *Sc*, *S. cerevisiae*; *Td*, *Torulapora delbrueckii*; *Op*, *Ogataea polymorpha*; *Ca*, *Candida albicans*, *Dh*, *Debaryomyces hansenii*; *Le*, *Lodderomyces elongisporus*; *Ba*, *Blastobotrys* (*Arxula*) *adeninivorans*; *Sp*, *Schizosaccharomyces pombe*; *Kl*, *Klyuveromuces lactis*; *Ls*, *Lipomyces strarkeyi*; *Ps*, *Pichia* (*Scheffersomyces*) *stipitis*, for which five putative *α*‐glucosidases, AGL1, MAL8, MAL7, MAL6 and MAL9, were included. ancMALS and ancMAL‐IMA refer to ancMALS (G279) and ancMAL‐IMA (A279)

*Op* is considered an early diverged yeast: in phylogenetic trees of Saccharomycotina it clusters with *Brettanomyces (Dekkera) bruxellensis*, *Komagatella (Pichia) pastoris*, *Candida albicans*, *Debaryomyces hansenii*, *Meyerozyma (Pichia) guillermondii* and *Scheffersomyces (Pichia) stipitis* (Curtin and Pretorius, 2014; Kunze *et al.*, 2014; Kurtzman *et al.*, 2015). Phylogenesis of these species can be tracked via the Mycocosm sequencing project and respective website (Grigoriev *et al.*, 2014). All the above‐listed yeasts diverged earlier from the main line of evolution leading to *Sc*, which has specialized *α*‐glucosidases (maltases and isomaltases) that presumably evolved from an ancestral promiscuous enzyme (Voordeckers *et al.*, 2012). For example, according to Hedges (2002), *Sc* and *C. albicans* have been separated for a very long time – about 840 million years. From the above‐mentioned phylogenetic group also harbouring *Op*, maltases have been purified and studied only from *C. albicans* (Williamson *et al.*, 1993) and *Op* (Alamäe and Liiv, 1998; Liiv *et al.*, 2001; and this study). The respective enzyme of *C. albicans* (*Ca*) hydrolyses maltose, sucrose (maltose‐like substrates) and *α*‐MG (an isomaltose‐like substrate), but it cannot use isomaltose (Geber *et al.*, 1992). Other substrates tested by us in current work were not addressed by Geber *et al.* (1992). The sequence of *Ca* enzyme characterized by the authors was retrieved and used for the alignment of *α*‐glucosidase sequences to create a phylogram (Figure 6). The signature amino acid sequence YTAGMVGEN of this protein reveals a Thr at the position corresponding to Val216 of IMA1 (Figures 3 and 4). The respective signature sequence of *Le* enzyme HTAGMVGDN is quite similar to that of *Op* maltase, differing from it only in two letters. This enzyme has also a Thr at the position equivalent to Val216 of IMA1 (Figure 4). Comparison of the substrate specificities of *Op* and *Le* proteins indicates that the *Le* protein uses isomaltose very poorly compared to *Op* maltase, whereas it uses another isomaltose‐like substrate, palatinose, perfectly well (Figure 3). Sequence identity between the *Le* and *Op* maltases is rather high, 59.8%, whereas identity of *Op* MAL1 with *Sc* MAL12 is 46.7% and with IMA1 47.3% (see supporting information, Table S3). Phylogenetic analysis of numerous sequences of yeast maltases and isomaltases by Voordeckers *et al.* (2012) showed that the *Le* and *Op* proteins cluster together, being distant from other proteins of the dataset.

Recently, *Brettanomyces bruxellensis* strains isolated from beer and wine were shown to grow on many *α*‐glucosides (Crauwels *et al*., 2015). Two genes for maltase (EIF45413.1 and EIF45252.1) and one for isomaltase (EIF45785.1) were detected in sequenced strains. One of the maltases (GenBank ID: EIF45413.1; Uniprot ID: I2JR11) has a signature amino acid sequence ITAGLVGDD, quite similar to that of *Op* MAL1 (FTAGLVGDN), yet overall identity between these enzymes is not high (~47%). This gene was found only in strains able to hydrolyse the *α*‐linked sugars maltose, turanose and melezitose as well as *α*‐MG (Crauwels *et al*., 2015). Inspection of genome sequences has disclosed multiple genes for *α*‐glucosidases in *Pichia (Scheffersomyces) stipitis* (Jeffries and Van Vleet, 2009; Brown *et al.*, 2010) and *D. hansenii* (Viigand *et al.*, 2005). Phylogenetic analysis of *α*‐glucosidase sequences (Figure 6) shows clustering of proteins from *P. stipitis*, *L. elongisporus*, *C. albicans*, *D. hansenii* and *O. polymorpha*.

A thorough paper on *Torulaspora pretoriensis* YK‐1 *α*‐glucosidase is available (Oda *et al.*, 1993). This protein used *α‐p*NPG, maltotriose, isomaltose, *α*‐MG and sucrose; it could also use maltose, but with poor affinity. Unfortunately, the whole sequence of the protein could not be retrieved, but according to the N‐terminal sequence presented in the paper, the enzyme could be identical or highly similar to the *T. delbrueckii* (*Td*) hypothetical isomaltase (GI: 367013896) we used in our paper in sequence alignments for Figures 4 and 6. BLAST search against the *Td* genome with *Op* maltase yielded three proteins with ~50% identity to MAL1. The *Td* enzyme is 83% identical to *Sc* IMA1, according to the protein sequence (see supporting information, Table S3), and the signature amino acid sequences of the two proteins are identical (Figure 4). In the phylogram (Figure 6), the *Td* enzyme and IMA1 of *Sc* cluster closely.

### *α*‐glucosidases of yeasts that diverged from the main evolution line of yeast earlier and their substrate specificity

*Saccharomyces* (belonging to the subphylum Saccharomycotina) is separated from *Schizosaccharomyces* (subphylum Taphrinomycotina) by about 1 Gya of evolutionary time, which is ~25% of the age of the Earth (Hedges, 2002). According to the Mycocosm database (Grigoriev *et al.*, 2014), from the Saccharomycotina, *Lipomyces starkeyi* (*Ls*) and *Blastobotrys adeninivorans* (*Ba*) are phylogenetically close to *Taphrinomycotina*. In the phylogram (Figure 6), *α*‐glucosidases of *Ls*, *Ba* and *Sp* are located distant from the other 15 proteins of the dataset, which are divided between the two clusters.

Kelly *et al.* (1985) reported that the extracellular *α*‐glucosidase from *Ls* had equally high activity on maltose and isomaltose; the activity on maltotriose and isomaltotriose was respectively 59% and 48% of that, and with *α‐p*NPG the respective activity was only 22%. Notably, this enzyme did not hydrolyse sucrose (Kelly *et al.*, 1985). An amylase has also been characterized from *Ls* (Kang *et al.*, 2004). This protein hydrolysed starch but not maltodextrins of G_2_–G_4_. Yet it formed G_2_ + G_3_ from G_5_, G_2_ + G_4_ or G_3_ + G_3_ from G_6_, and G_3_+ G_4_ from G_7_ glucans. Also, the enzyme was inhibited by acarbose (Kang *et al.*, 2004). Unfortunately, the sequence of the *Ls α*‐glucosidase protein is not presented in the paper by Kelly *et al.* (1985) and we could not find it from other databases. BLAST search of the genomic sequence of *Ls* NRRL Y–11557 with *Op* maltase sequence as a query yielded numerous putative *α*‐glucosidase genes, but their functionality and the substrate specificity of respective proteins remains unknown. The most similar to *Op* MAL1 protein (48.7% identity) was used by us in sequence alignments and phylogeny analyses (Figures 4 and 6). According to its signature amino acid sequence YTVNKLSGE (Figure 4), the *Ls* enzyme may have a wide substrate range. At the same time, the *Ls* genome also encodes putative *α*‐glucosidases that have a Val at the position equivalent to Val216 of IMA1, and that may thus be more similar to isomaltases (data not shown). The genome of *Blastobotrys (Arxula) adeninivorans* (Kunze *et al.*, 2014) also revealed the presence of an *α*‐glucosidase gene which may encode isomaltase, as it has a Val at the position corresponding to Val216 of IMA1 (Figure 4). The sequence identity of this predicted protein with *Op* MAL1 is 42.7% (see supporting information, Table S3). Few papers are available on *α*‐glucosidases of *Sp*. For example, the extracellular hyperglycosylated *α*‐glycosidase of *Sp* AHU 3719 preferred short substrates, e.g. maltose and maltotriose, to longer ones and hydrolysed aside of *α‐*1,4 also *α‐*1,2; *α‐*1,3 and *α‐*1,6‐glycosidic linkages. The activity of this protein was highest with maltotriose, but maltose and maltotetraose were also good substrates. The authors showed that the *Sp* acted only poorly on soluble starch and that its catalytic site comprised three subsites (Okuyama *et al.*, 2005). As in case of the *Ls* enzyme (Kelly *et al.*, 1985), *α‐p*NPG was a rather poor substrate for the *Sp* enzyme (Okuyama *et al.*, 2005). Poor hydrolysis of *α‐p*NPG compared to other *α*‐glucosidic substrates is also characteristic for bacterial maltases (Schönert *et al*., 1998; Egeter and Brückner, 1995). Hydrolysis of sucrose was not assayed by Okuyama *et al.* (2005). The MAL1 protein of *Sp*, with 44.1% of sequence identity with *Op* MAL1, was included by us to the alignment and phylogenetic assay of yeast *α*‐glucosidases. This protein was heterologously expressed in *E. coli* and studied by Chi *et al.* (2008). Most probably, the MAL1 of *Sp* is intracellular. The best substrates of the *Sp* MAL1 protein were *α‐p*NPG and maltose; it also acted on soluble starch and dextrin and had some activity with sucrose (Chi *et al.*, 2008). Jansen *et al.* (2006) reported on the extracellular maltase of *Sp* CBS 356, which was rather specific for maltose, e.g. it did not act on maltotriose and turanose and showed only low activity on *α‐p*NPG. The ability of *Sp* 972 h^–^ to grow on various *α*‐glucosides was tested by us in comparison to *Op* and is presented in Table 4.

### Early phylogenetic history of maltases and isomaltases

This subject has been thoroughly addressed by Gabrishko (2013). Analysis of 103 putative *α*‐glucosidase proteins encoded in 37 fungal genomes suggested that the common ancestor of the Ascomycota had two *α*‐glucosidase‐encoding genes, and that in the subphylum Saccharomycotina one of these genes (the gene A, coding for isomaltase) was lost and the other one (the gene B, coding for maltase) had further lineage‐specific duplication. According to Gabrishko (2013), isomaltase‐type specificity evolved repeatedly and independently in distinct lineages of the Saccharomycotina. Phylogenetic assay of fungal subtelomeric gene families, including yeast *MALT* (transporter), *MALS* (maltase and isomaltase) and *MALR* (regulator) genes (Brown *et al.*, 2010), indicated that the common ancestor of yeasts addressed in the study (*S. cerevisiae*, *Candida glabrata*, *K. lactis*, *P. stipitis*, *D. hansenii*, *Y. lipolytica* and *Sz. pombe*) had only few *MAL* genes, which were completely lost in some lineages and expanded in others. For example, in *K. lactis* two *MALT* and *MALS* genes were detected, but *MALR* was absent, *Y. lipolytica* had a *MALR* and *MALT* gene, but no *MALS* gene, whereas *P. stipitis* had five genes in each family (Brown *et al.*, 2010). We compared sequences of the five MALS proteins (MAL8, MAL7, MAL6, MAL9 and AGL1) (Jeffries and Van Vleet, 2009; Brown *et al.*, 2010) of *P. stipitis* with the respective sequences of other yeasts (see supporting information, Table S3). The *P. stipitis* (*Ps*) proteins were 59.7–61.8% identical to *Op* MAL1 (see Table S3) and their signature amino acid sequences were almost identical with that of MAL1 (FTAGLVGDN), except for the position shown underlined. In *Ps* proteins, either N, T, W or E resided at that position (data shown shown). The phylogram in Figure 6 indicates that *Ps α*‐glucosidases cluster closely with *Op* MAL1. We predict that the *Ps* MAL8, MAL7, MAL6, MAL9 and AGL1 proteins may be promiscuous *α*‐glucosidases similar to *Op* MAL1. It would be interesting to study the substrate specificity pattern of these proteins. As suggested in Brown *et al.* (2010), multiple *MALS* genes of *Ps* resulted from repeated gene duplications in the lineage. We hypothesize that *Op* MAL1 may be similar to the ancestor of *Ps α*‐glucosidases, or that they may have a common ancestor. Considering the data on evolution of *α*‐glucosidases by Gabrishko (2013), MAL1 of *Op* has probably been inherited from a common ancestor of Ascomycota and has evolved to use isomaltose‐like substrates.

### Growth of *Op* wild‐type strain and deletants of maltase and *α*‐glucoside permease genes on substrates of the *Op* maltase protein

Table 4 shows that wild‐type *Op* does not grow on *α*‐MG and soluble starch. Growth on trehalose (a disaccharide with *α‐*1,1‐glycosidic linkage between the two glucose residues) was also presented, because trehalose is transported into *Op* by MAL2 permease (Viigand and Alamäe, 2007), but inside the cell it is hydrolysed not by maltase but a trehalase (Liiv *et al.*, 2001; Viigand and Alamäe, 2007). Table 4 confirms these data, as a maltase deletant did grow on trehalose but a permease deletant did not. As neither maltase deletant nor *α*‐glucoside permease deletant could grow on maltose, sucrose, turanose, maltotriose, maltulose, melezitose, isomaltose, palatinose and isomalto‐oligosaccharides, maltase and *α*‐glucoside permease are equally required for the utilization of these sugars. In this study we showed that all these substrates are hydrolysed by MAL1. The growth experiment (Table 4) additionally showed that the *α*‐glucoside permease MAL2 of *Op* transports all the above‐mentioned *α*‐glucosides into the cell. An early diverged yeast, *Sp*, was used for comparison in the growth experiment. This yeast, which has more than one enzyme for the utilization of *α*‐glucosides, grew on all tested substrates except for melezitose, trehalose and soluble starch.

**Table 4:** Growth of wild‐type *O. polymorpha* and mutants with deleted maltase (*ΔMAL1*) or *α*‐glucoside permease genes (*ΔMAL2*) on solid medium supplemented with substrates that are hydrolysed by the maltase of *O. polymorpha*. *Sz. pombe* strain 972 h^–^ was studied for comparison; growth was evaluated on day 5 in the case of *Op* and on day 11 in the case of *Sp*

Substrate	*Op* WT	*Op* ΔMAL1	*Op* ΔMAL2	*Sp*
Maltose	+	–	–	+
Sucrose	+	–	–	+
Turanose	+	–	–	+
Maltotriose	+	–	–	+
Maltulose	+	–	–	+
Melezitose	+	–	–	–
*α*‐MG	–	–	–	+
Isomaltose	+	–	–	+
Palatinose	+	–	–	+
Trehalose	+	+	–	–
Soluble starch	–	–	–	–
Isomalto‐oligosaccharides	+	–	–	+

### Substrates for maltases and isomaltases in nature, now and hundreds of millions of years ago

Nowadays, the majority of maltose, isomaltose and other substrates for *α*‐glucosidases are probably produced from the hydrolysis of starch by microbial amylases (Janeček, 2009). Glycogen is present in organisms from all three domains of life, eukaryotes, bacteria and archaea (Ball *et al*., 2011), and its degradation also yields products for both maltases and isomaltases. Most interestingly, even thermophilic archaea have *α*‐amylases and pullulanases that can degrade glycogen‐like polymers to products used by maltases and isomaltases (Janecek *et al.*, 1999; Lévêque *et al*., 2000). Many current‐day plants (sugar cane, sugar beet) are specifically rich in sucrose (Winter and Huber, 2000). Sucrose is also synthesized by cyanobacteria and proteobacteria (Lunn, 2002), meaning that this sugar was available on Earth before the emergence of eukaryotes. Most ancient land plants, liverworts and mosses, living on Earth several hundreds of millions of years ago (Hedges, 2002) also synthesize sucrose (Galloway and Black, 1989). Many organisms, including plants, yeasts, filamentous fungi, bacteria and even insects, can transform sucrose to other products. For example, palatinose is produced as a sucrose isomerization product by species from several *Proteobacteria* genera, such as *Klebsiella*, *Pseudomonas*, *Pantoea* and *Serratia* (Lee *et al.*, 2011 and references therein). Kestoses are produced from sucrose by microbial levansucrases, invertases and inulosucrases (Visnapuu *et al.*, 2015 and references therein). In the normal habitat of *Op*, substrates for MAL1 may originate from the hydrolysis of starch and glycogen, but may also arise from sucrose isomerization by enzymes of various organisms. *Ogataea* strains have been isolated from soil, spoiled orange juice, the leaf surfaces and exudates of plants, insect guts and other places (Morais *et al.*, 2004; Limtong *et al.*, 2008 and references therein). These habitats provide methanol from the degradation of pectin and lignin (Nakagawa *et al.*, 2005), but may also contain substrates for *α*‐glucosidases. For example, two *Ogataea* species described in Limtong *et al.* (2008) grew on maltose, melezitose and *α*‐MG, thus being similar to *Op*.

## Concluding remarks

Our study shows that *O. polymorpha*, which diverged earlier from the main line of evolution leading to *Saccharomyces* spp., possesses a promiscuous MAL1 enzyme. This *α*‐glucosidase protein can use both maltose‐ and isomaltose‐like sugars, thus being similar to the resurrected hypothetical ancestor of specialized enzymes – maltases and isomaltases – of *Saccharomyces* and other ‘modern’ yeasts. This fact strongly supports the hypothesis raised by Voordeckers *et al.* (2012). Based on phylogenetic analysis, we assume that the *α*‐glucosidases encoded in the genome of *Pichia (Scheffersomyces) stipitis* may also be similar to *Op* MAL1, according to the substrate specificity. In MAL1 of *Op*, the maltase and isomaltase activities are accommodated in one enzyme and these activities can be separately optimized. The MAL1 enzyme can be mutated both ways, to increase either its maltase‐ or isomaltase‐hydrolysing abilities. MAL1 has good stability and should be considered a suitable candidate for co‐crystallization with substrates to determine the structure of a promiscuous *α*‐glucosidase of yeast origin. In addition, although the MAL1 protein of *O. polymorpha* has been referred to as ‘maltase’ in publications since 2001 (Liiv *et al*., 2001), it should be considered ‘maltase–isomaltase’ according to substrate specificity.

## Supporting information


**Figure S1**. Structures of *Op* maltase substrates *α*‐methylglucoside (methyl *α*‐d‐glucopyranoside; *α*‐MG) and *p*‐nitrophenyl‐*α*‐d‐glucopyranoside (*α‐p*NPG)
**Figure S2.** *Op* maltase does not hydrolyse nystose.
**Table S1**. Primers used for cloning and site‐directed mutagenesis of *Ogataea polymorpha* maltase gene *MAL1*.
**Table S2**. Abbreviations and Accession Nos of 18 *α*‐glucosidases of various yeast species addressed in this study.
**Table S3**. Identity matrix of protein sequences of 18 *α*‐glucosidases addressed in this study.

Supporting info item
