# Supplementary material for: Maltase protein of Ogataea (Hansenula) polymorpha is a counterpart to the resurrected ancestor protein ancMALS of yeast maltases and isomaltases
Source: Yeast. 2016 Apr 21;33(8):415–32. doi: 10.1002/yea.3157 (PMC5074314; doi:10.1002/yea.3157)
Supplement: Supplementary file 1 — Supporting info item [file YEA-33-415-s001.pdf]

Supporting information

Maltase protein of *Ogataea (Hansenula) polymorpha* is a counterpart to resurrected ancestor protein ancMALS of yeast maltases and isomaltases

Katrin Viigand[1], Triinu Visnapuu[1,2], Karin Mardo, Anneli Aasamets and Tiina Alamäe[*]

Department of Genetics, Institute of Molecular and Cell Biology, University of Tartu, Riia 23, 51010 Tartu, Estonia

[1] These authors contributed equally to this work

[2] Current address: Department of Systems Biology, Technical University of Denmark, Elektrovej, building 375, 2800 Kgs. Lyngby, Denmark

[*] Correspondence to: T. Alamäe, Department of Genetics, Institute of Molecular and Cell Biology, University of Tartu, Riia 23, 51010 Tartu, Estonia

Tel.: +372 7375013

E-mail: talamae@ebc.ee

Table S1. Primers used for cloning and site-directed mutagenesis of *Ogataea polymorpha* maltase gene *MAL1*. The underlined sequences are pairing with the pURI3Cter vector (Curiel *et al*., 2011) and sequences complementary to the beginning and end of the *MAL1* gene are shown in italic. Start and stop codons are marked with bold letters. Mutated codons are highlighted with a gray background.

Primer and function	Sequence	Changed codon and amino acid	Reference
MAL1_PURICterm_Fw<br>Cloning Fw primer	5'-<u>TAACTTTAAGAAGGAGATATACAT</u>***ATG****ACTATCGAGTCTCAAGAACCT*-3'	No change	This work
MAL1_PURICterm_Rev<br>Cloning Rev primer	5'-<u>GCTA**TTA**ATGATGATGATGATGATG</u>*ATTGACCTCGATCAGTCTACCTTC*-3'	No change	This work
T7<br>Mutagenesis Fw primer	5'-TAATACGACTCACTATAGGG-3'	No change	Viigand *et al*., 2005
Asp199AlaRev<br>Mutagenesis Rev primer	5'-AGGCCGGCAGTGGCGATTCTG-3'	GTC → GGC;<br>Asp → Ala	This work
Thr200ValRev<br>Mutagenesis Rev primer	5'-GAGGCCGGCAACGTCGATTCTG-3'	AGT → AAC;<br>Thr → Val	This work

Table S2. Abbreviations and accession numbers of 18 α-glucosidases of various yeast species addressed in the work.

**Abbreviation**	**Yeast species and enzyme**	**UniProtKB accession number**
Ba	*Blastobotrys (Arxula) adeninivorans* CBS8244 α-glucosidase*	A0A060T069
Sp	*Schizosaccharomyces pombe* 972 h- ATCC24843 α-glucosidase	Q9P6J3
Kl	*Kluyveromyces lactis* NRRLY-1140 α-glucosidase	Q6CSK8
*Sc*_MAL12	*Saccharomyces cerevisiae* Sc288c ATCC204508 maltase MAL12	P53341
ancMALS	Resurrected ancestral maltase	(Voordeckers *et al*., 2012)
ancMAL-IMA	Resurrected ancestral maltase-isomaltase	(Voordeckers *et al*., 2012)
*Sc*_IMA1	*Saccharomyces cerevisiae* Sc288c ATCC204508 isomaltase IMA1	P53051
Td	*Torulaspora delbrueckii* CBS1146 isomaltase*	Q5J9B3
Ls	*Lipomyces starkeyi* CBS1807 (NRRLY-11557) α-glucosidase*	Protein ID: 3262; scaffold_6:175313-177164
Op	*Ogataea (Hansenula) polymorpha* CBS4732 α-glucosidase MAL1	Q9P8G8
Le	*Lodderomyces elongisporus* CBS2605 (NRRL YB-4239) α-glucosidase	A5DVH3
*Ps*_AGL1	*Pichia (Scheffersomyces) stipitis* CBS6054 α-glucosidase AGL1*	A3LWN5
*Ps*_MAL8	*Pichia (Scheffersomyces) stipitis* CBS6056 maltase MAL8*	A3LXA2
*Ps*_MAL7	*Pichia (Scheffersomyces) stipitis* CBS6057 maltase MAL7*	A3LUP5
*Ps*_MAL6	*Pichia (Scheffersomyces) stipitis* CBS6055 maltase MAL6*	A3LNM1
Ca	*Candida albicans* NCBI5476 (ATCC18804) maltase	Q02751
*Ps*_MAL9	*Pichia (Scheffersomyces) stipitis* CBS6054 maltase MAL9*	A3GIC0
Dh	*Debaryomyces hansenii* CBS767 (NRRLY-7426) α-glucosidase*	Q6BXY6

* Predicted from the gene sequence, experimentally not studied

Table S3. Identity matrix of protein sequences of 18 α-glucosidases addressed in the work. The matrix was calculated using MUSCLE (v. 3.8.31) and created using Clustal2.1 as alignment format. Identity values are shown in percentages (%). For abbreviations see Table S2.

	Ba	*Sp*	*Kl*	*Sc*_MAL12	ancMALS	ancMAL-IMA	*Sc*_IMA1	*Td*	*Ls*	*Op*	*Le*	*Ps*_AGL1	*Ps*_MAL8	*Ps*_MAL7	*Ps*_MAL6	*Ca*	*Ps*_MAL9	*Dh*
Ba	100.0	58.7	40.8	43.0	43.9	43.9	42.9	41.9	46.0	42.7	43.5	42.4	45.1	43.7	45.5	44.9	44.6	43.5
Sp	58.7	100.0	39.1	40.3	42.7	42.7	39.8	40.0	47.1	44.1	44.4	44.6	46.5	45.3	46.7	45.8	45.0	46.7
Kl	40.8	39.1	100.0	62.2	71.0	68.6	61.2	62.8	46.6	48.5	51.2	51.6	50.4	52.0	51.2	49.1	50.4	49.1
*Sc*_MAL12	43.0	40.3	62.2	100.0	81.3	84.9	71.8	70.5	46.0	46.7	49.2	51.2	49.6	50.6	51.3	49.6	50.7	50.8
ancMALS	43.9	42.7	71.0	81.3	100.0	95.6	78.4	76.2	47.9	50.2	53.8	53.8	54.5	54.7	55.2	51.5	51.9	53.3
ancMAL-IMA	43.9	42.7	68.6	84.9	95.6	100.0	82.2	78.9	47.9	49.1	52.9	53.1	53.1	53.5	53.8	50.3	51.6	52.9
*Sc*_IMA1	42.9	39.8	61.2	71.8	78.4	82.2	100.0	83.0	46.8	47.3	51.2	51.0	51.9	50.4	52.2	49.7	51.6	52.0
Td	41.9	40.1	62.8	70.5	76.2	78.9	83.0	100.0	47.7	45.7	50.4	51.2	50.8	50.1	50.6	49.6	49.1	52.0
Ls	46.1	47.1	46.6	46.0	47.9	47.9	46.8	47.7	100.0	48.7	51.6	53.4	52.5	53.7	53.9	52.1	53.3	56.0
Op	42.7	44.1	48.5	46.7	50.2	49.1	47.3	45.7	48.7	100.0	59.8	60.9	61.5	61.8	61.8	58.9	59.7	62.8
Le	43.5	44.4	51.2	49.2	53.8	52.9	51.2	50.4	51.6	59.8	100.0	66.2	69.7	68.8	70.1	66.1	64.4	69.0
*Ps*_AGL1	42.4	44.6	51.6	51.2	53.8	53.1	51.0	51.2	53.4	60.9	66.2	100.0	76.9	76.4	78.9	65.1	66.6	70.1
*Ps*_MAL8	45.1	46.5	50.4	49.6	54.5	53.1	51.9	50.8	52.5	61.5	69.7	76.9	100.0	78.7	82.7	65.7	67.0	71.6
*Ps*_MAL7	43.7	45.3	52.0	50.6	54.7	53.5	50.4	50.1	53.7	61.8	68.8	76.4	78.7	100.0	85.5	63.6	68.7	71.7
*Ps*_MAL6	45.5	46.7	51.2	51.3	55.2	53.8	52.2	50.6	53.9	61.8	70.1	78.9	82.7	85.5	100.0	65.1	68.0	72.4
Ca	44.9	45.8	49.1	49.6	51.5	50.3	49.7	49.6	52.1	58.9	66.1	65.1	65.7	63.6	65.1	100.0	70.7	68.0
*Ps*_MAL9	44.6	45.0	50.4	50.7	51.9	51.6	51.6	49.1	53.3	59.7	64.4	66.6	67.0	68.7	68.0	70.7	100.0	72.8
Dh	43.5	46.7	49.1	50.8	53.3	52.9	52.0	52.0	56.0	62.8	69.0	70.1	71.6	71.7	72.4	68.0	72.8	100.0

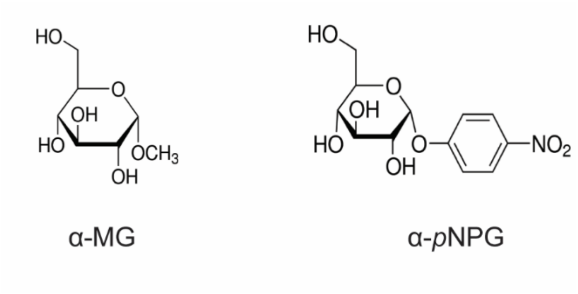

Figure S1. Structures of *Op* maltase substrates α-methylglucoside (methyl α-D-glucopyranoside; α-MG) and *p*-nitrophenyl-α-D-glucopyranoside (α-*p*NPG)

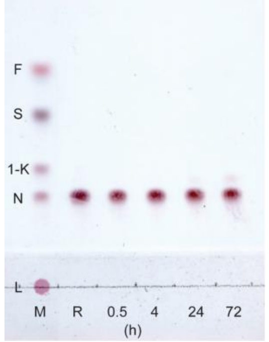

Figure S2. The *Op* maltase does not hydrolyze nystose. Reaction was conducted with 50 mM nystose in 100 mM K-phosphate buffer (pH 6.5) containing 0.1 mM EDTA using 13 µg/ml of *Op* maltase protein (1 U/ml) and at shown time points the samples were taken out, the enzyme was inactivated by heating (5 min, 96°C) and the aliquots (0.5 µl) were analysed on TLC. Sugar marker (M): fructose, F; sucrose, S; 1-kestose, 1-K; nystose, N; levan, L. Reference (R) designates 50 mM nystose which was incubated without the maltase added.

Supplementary references

Curiel JA, de las Rivas B, Mancheño JM, Muñoz R. (2011). The pURI family of expression vectors: A versatile set of ligation independent cloning plasmids for producing recombinant His-fusion proteins. *Protein Expr Purif* **76:** 44-53.

Viigand K, Tammus K, Alamäe T. (2005). Clustering of *MAL* genes in *Hansenula polymorpha*: Cloning of the maltose permease gene and expression from the divergent intergenic region between the maltose permease and maltase genes. *FEMS Yeast Research* **5:** 1019-1028.

Voordeckers K, Brown CA, Vanneste K, *et al.* (2012). Reconstruction of ancestral metabolic enzymes reveals molecular mechanisms underlying evolutionary innovation through gene duplication. *PLoS Biol* **10:** e1001446.
